# Meleney’s gangrene triggered by suprapubic catheter dislodgment and urine extravasation: A case report and review of management strategies

**DOI:** 10.5339/qmj.2026.67

**Published:** 2026-09-22

**Authors:** Ahmed Mohammed Umar, Mohammed Mustapha Kura, Daniel Joshua Maina, Abdullahi Khalid, Ayodeji Olawale Afolayan

**Affiliations:** 1 Department of Surgery, Gombe State University, Gombe State, Nigeria; 2 Department of Surgery, Federal Teaching Hospital Gombe, Gombe State, Nigeria; 3 TetFund Centre of Excellence in Urology & Nephrology, Institute of Urology & Nephrology, Usmanu Danfodiyo University and Teaching Hospital, Sokoto, Nigeria

**Keywords:** Meleney’s gangrene, necrotizing fasciitis, necrotizing soft tissue infection, suprapubic cystostomy, Nigeria.

## Abstract

**Background:**

Necrotizing soft tissue infection represents a spectrum of bacterial infections that result in rapidly progressive, life-threatening tissue necrosis. Early diagnosis remains a challenge due to the rarity of the condition and variable presentation. Herein, we present a case of Meleney’s gangrene managed at a tertiary healthcare facility in Nigeria.

**Case Presentation:**

The patient presented to the emergency department with a 13-hour history of inability to pass urine associated with pain. Physical examination revealed a tender urinary bladder consistent with a diagnosis of acute urinary retention. Following multiple failed attempts at urethral catheterisation, an ultrasound-guided percutaneous suprapubic cystostomy (SPC) was performed. Four days after being discharged home, he came back with a complaint of a dislodged suprapubic tube and leakage of urine through the SPC stoma. Physical examination revealed mild peristomal skin discolouration and tenderness. He underwent an open suprapubic cystostomy. However, the skin discoloration progressed to Meleney’s gangrene. Surgical debridement was performed, and broad-spectrum antibiotic therapy was initiated. The wound was later skin grafted after satisfactory wound care. However, most of the graft was lost, and the patient had successful secondary wound healing.

**Discussion:**

We strongly believe that the dislodgement of the suprapubic tube and subsequent extravasation of urine into the tissue planes and the retropubic space were the likely precipitating events in our patient, especially given that the urine was infected. Chemicals in urine, such as urea and ammonia, can irritate tissue, leading to chemical cellulitis and inflammation. Additionally, the extravasated urine creates an ideal environment for microbial proliferation. Consequently, the combined chemical and bacterial assault potentiates the local inflammatory response, allowing infection to spread quickly along fascial planes. Successful treatment relies on surgical debridement, broad-spectrum antibiotic therapy, wound management, and supportive care.

**Conclusion:**

This case report underscores the importance of proper suprapubic catheter placement to avoid dislodgement and urinary leak into subcutaneous tissue planes, which can trigger Meleney’s gangrene. It provides a clear lesson that any suprapubic tube dislodgement must be regarded as high risk, warranting vigilant monitoring for signs of deep soft tissue infection.

## 1. INTRODUCTION

Necrotizing soft tissue infections are fulminant infections involving any of the layers of soft tissues from the dermis up to the deep fascia or muscle with associated extensive necrosis and systemic toxicity, irrespective of anatomical site or depth of tissue involvement.^1^ These encompass not just Meleney’s gangrene but also necrotizing cellulitis, Fournier’s gangrene, necrotizing myositis, and myonecrosis.^1^ These infections can occur on any part of the body but are most commonly reported in the extremities, perineum, and genitalia, while occurrence on the abdomen and chest is rare.^1^ Risk factors include diabetes mellitus, obesity, immunosuppression, and peripheral vascular disease.^1,2^ Increasing age is also associated with an increased incidence of necrotizing fasciitis, and higher mortality is observed in this category of patients.^3^ The incidence of necrotizing soft tissue infection appears to be on the rise, possibly due to increased awareness of the disease process and earlier recognition of the disease.^2^ However, mortality remains high even though recent evidence suggests that it has decreased to a rate of between 10% and 20%, with a wide range across series.^2^

Meleney’s gangrene is a form of necrotizing soft tissue infection that involves the skin, subcutaneous tissues, and muscle fascia of the anterior abdominal wall.^4^ As a necrotizing fasciitis, Meleney’s gangrene is also known as progressive bacterial synergistic gangrene. It is lethal, with a mortality rate approaching 40%, but it can be higher in immunocompromised and diabetic patients.^5^ The severity of the disease is believed to be related to the synergistic effects of multiple bacteria, and it often manifests after surgery or even minor trauma.^6^ The main culprit is the synergistic interaction between haemolytic aerobic Staphylococcus aureus and microaerophilic Streptococcus.^7^ As with any form of necrotising soft tissue infection, survival depends on early institution of management with broad-spectrum antibiotics, early surgical debridement, and transfer to a tertiary care centre.^2^

In this report, we discussed the management of a man with Meleney’s gangrene at Federal Teaching Hospital, Gombe, Nigeria. This is a tertiary health facility and a major referral centre in the northeast of Nigeria.^8^ Written informed consent was obtained from the patient, and institutional approval was waived as this is not required for publishing a case report in our institution.

## 2. CASE PRESENTATION

A 45-year-old man with no medical co-morbidity presented to the emergency room with a thirteen-hour history of inability to pass urine associated with suprapubic swelling and pain. There was a preceding history of progressive worsening voiding and storage lower urinary tract symptoms for about a year following a urethral injury from a road traffic accident. On examination, the patient was in pain, and the abdominal examination revealed a palpable and tender urinary bladder in keeping with acute urinary retention.

After three failed attempts at urethral catheterization, presumably due to urethral stricture, the patient was counselled for percutaneous suprapubic cystostomy (PSC). Under sterile conditions, an ultrasound-guided SPC was performed, with passage of a 22F Foley catheter that was inflated with 15ml of sterile water. Approximately 700ml of amber-coloured, foul-smelling urine was drained and sent for microscopy, culture, and sensitivity testing. He also received intravenous levofloxacin, 500mg daily for 48 hours, following which he was discharged home on oral levofloxacin, with postoperative instruction on catheter care. However, he came back to the emergency department 4 days later with a complaint of urine leak through the stoma. He reported that the suprapubic tube fell off the day he was discharged. Abdominal examination revealed mild discolouration and tenderness of the skin around the stoma, with continuous urine leakage. The urine culture yielded Escherichia coli that was sensitive to Ceftriaxone. Peri-stomal cellulitis was diagnosed. Intravenous Ceftriaxone was commenced, and he subsequently underwent open suprapubic cystostomy. Postoperatively, the antibiotic was continued; however, the skin discoloration rapidly progressed to bullae formation, which ruptured, discharging foul-smelling pus after 5 days. He was febrile (38.9°C) with a pulse rate and blood pressure of 102 beats per minute and 100/70 mmHg, respectively.

Abdominal examination revealed lower abdominal wall gangrene measuring 22cm x 12cm, as shown in Figure 1A. His full blood count showed a packed cell volume of 33% and leucocytosis of 14 x 10^9^/L. His serum potassium was 2.8 mmol/L, which was corrected using parenteral potassium chloride. His fasting blood sugar was normal, and his retroviral screening was non-reactive. A diagnosis of Meleney’s gangrene was established. Intravenous Gentamicin and Metronidazole were added to his treatment regimen. Similarly, he received 0.5ml of tetanus toxoid and intravenous normal saline. He subsequently had surgical debridement of the gangrene, drainage of the retropubic space, and revision of the suprapubic cystostomy. Intraoperative findings were extensive gangrene involving the skin, subcutaneous tissues, and part of the rectus abdominis muscles with a purulent collection in the retropubic space. The peritoneum and the urinary bladder were covered by necrotic slough as shown in Figure 1B.

The intravenous antibiotics were continued postoperatively for 72 hours and later converted to oral antibiotics (Ofloxacin), based on the culture sensitivity profile. Culture of the debrided tissue yielded Staphylococcus aureus and Escherichia coli. Oral Metronidazole was continued, and wound dressing was performed using normal saline and pure honey. Despite the progressive improvement of the wound, the purulent discharge from the retropubic space continued up to 14 days post-debridement, as shown in Figure 1C. Negative pressure wound therapy (NPWT) was instituted, resulting in improved granulation, resolution of the purulent discharge, and wound contraction, as shown in Figure 2A. The wound condition was ideal for skin grafting after 28 days of intensive wound care. The wound was skin grafted as shown in Figure 2B, but most of the graft was lost. The patient declined further skin grafting and opted for secondary wound healing. He was discharged home, having spent 36 days on admission. The patient’s management was continued on an outpatient basis. The wound progressively contracted and healed, with the patient reporting satisfaction.

## 3. DISCUSSION

This case report aims to highlight a rare and potentially life-threatening instance of a progressive bacterial synergistic gangrene following suprapubic tube dislodgement and subsequent urinary extravasation. By detailing this unusual etiology, this report aims to emphasize the importance of early clinical recognition, a high index of suspicion for atypical soft tissue infections, and the importance of prompt, aggressive surgical debridement and multidisciplinary management to improve patient survival.

Despite being a safe procedure, complications from suprapubic cystostomy, such as bleeding, infection, and obstruction, have been reported.^9^ Less frequent complications include bowel perforation, enterocutaneous fistula, catheter migration, or knotting, among others.^9^ Even though Meleney’s gangrene is frequently reported following intra-abdominal surgery near the surgical wound, there is a paucity of reports of a dislodged suprapubic tube leading to Meleney’s gangrene. Surgeons must have a high index of suspicion because patient survival depends on expedient management with broad-spectrum antimicrobials, early surgical debridement, and supportive care.^2,10^

To our knowledge, Meleney’s gangrene has not been reported as a complication of SPC. Meleney’s gangrene may perhaps occur following SPC due to the creation of the SPC on infected skin, non-sterile conditions, or the seepage of urine into tissue planes. We strongly believe that the precipitating event in our index case was the dislodgement of the suprapubic tube and urine leakage into the tissue planes and retropubic space, particularly given that the urine was infected. This is because chemicals such as urea and ammonia in urine can irritate tissues, leading to chemical cellulitis and inflammation. Furthermore, the extravasated urine provides an ideal medium for microbial proliferation. Therefore, the combined chemical and bacterial assault can exacerbate the local inflammatory response and promote rapid progression of infection along fascial planes.

The disease severity is linked to the virulence related to the synergy of multiple pathogens in the affected area.^11^ Even though the infection is mostly polymicrobial (type I), it can also be monomicrobial (type II), which typically involves group A beta-haemolytic Streptococcus and, less often, Staphylococcus aureus.^12^ Meleney’s gangrene may be associated with severe pain due to thrombosis of the cutaneous vessels leading to gangrene.^11^ Early features include erythema or skin discolouration caused by the subtle changes happening underneath that have not manifested.^2^ At this phase, it is often difficult to differentiate a necrotising infection from cellulitis.^12^ This leads to misdiagnosis or delayed diagnosis, as seen in our index patient. The evolution of necrotising soft tissue infection is usually very rapid, in contrast to cellulitis or abscess.^10^ The most reliable feature is pain, often out of proportion to the examination findings.^2,12^ Other skin manifestations include bullae, ecchymosis, and anaesthesia. These findings usually precede skin necrosis but occur in a minority of patients in the later stages of the disease.^2^ Systemic signs include fever and tachycardia, while in severe infection, hypotension and shock can occur.^13^

The diagnosis is clinical, but radiological investigation may provide valuable information in doubtful situations. Diffuse thickening of the subcutaneous tissue on ultrasonography, with fluid collection over 4mm along the deep fascial layer, is highly suggestive of necrotising fasciitis.^13^ A plain X-ray is not recommended as a screening tool for necrotising soft tissue infection.^13^ However, a computed tomography (CT) scan finding of fascial thickening, gas in soft tissues, fat stranding, and fluid in the involved tissues is consistent with necrotising soft tissue infection.^10,13^ Laboratory investigations include full blood count, C-reactive protein, blood sugar, serum electrolytes, urea, and creatinine. Similarly, diabetes mellitus and other immunosuppressive conditions, including retroviral disease, should be ruled out.

The principles of management of Meleney’s gangrene are early establishment of diagnosis, fluid resuscitation, broad-spectrum antibiotic therapy, source control, pain management, wound care, and adjuvant therapy. Resuscitation entails fluid and electrolyte replacement and blood therapy where indicated. Broad-spectrum antimicrobials that cover gram-negative, gram-positive, and anaerobic organisms are recommended.^2^ Infected fluids should be drained, and infected soft tissues should be adequately debrided. The promptness and completeness of the debridement significantly influence patient outcomes.^13^ Multiple debridements may be needed to achieve source control. All fascial layers should be opened to explore the underlying muscle for viability, colour, and contractility while debriding tissues that appeared ischaemic or necrotic.^2^ The debrided tissue should be sent for microbiological investigation.

Pure honey is a useful adjunct in post-debridement wound care due to its antimicrobial, debriding, anti-inflammatory, and pro-healing properties.^14^ Its antimicrobial effects are attributable to its high osmolarity, low water activity, and acidity.^14^ Additionally, honey contains compounds such as bee defensin-1 peptide, phenolic compounds, hydrogen peroxide, and methylglyoxal that directly inhibit microbial growth and survival.^14^ It exerts its anti-inflammatory effects through various mechanisms, including inhibiting tumor necrosis factor alpha expression and decreasing pro-inflammatory cytokine levels by attenuating nuclear factor kappa B.^14^ It also reduces the activity of cyclooxygenase 1 and 2, which are essential for prostaglandin synthesis.^14^ Lastly, it provides a moist wound environment, promoting autolytic debridement. Rai et al.^15^ compared topical honey with Edinburgh University Solution of Lime in managing necrotizing fasciitis wounds and observed that honey promotes faster wound healing and shorter hospital stays.

Pain management is a vital and integral component of patient management. It improves patient cooperation during wound care, as wound dressing can be very painful. Multimodal pain management may be needed to achieve satisfactory pain control.^2^ The use of hyperbaric oxygen (HBO) is one of the adjunct therapies in managing Meleney’s gangrene.^13^ This involves delivering 100% oxygen at a pressure of 2-3 absolute atmospheres.^13^ The goal is to increase the concentration of dissolved oxygen in the blood, resulting in higher tissue oxygen tension.^13^ The beneficial effects include improved leukocyte function, inhibition of anaerobic growth, suppression of toxin production, and enhanced antibiotic effectiveness.^13^ Although HBO can be helpful if available, it should not delay or interfere with standard treatment.^13^

Despite studies demonstrating better resolution of organ dysfunction with the adjuvant use of intravenous immunoglobulin, evidence is conflicting regarding its benefit in group A Streptococcus.^2^ Negative pressure wound therapy (NPWT) can be used for wound care after the complete removal of necrotic tissue to promote healing.^13^ Sub-atmospheric pressure is applied in NPWT either continuously or intermittently to improve blood flow and reduce tissue edema, thereby increasing tissue perfusion.^13^ It also helps absorb exudates and fluids, inhibits infection, and encourages the migration of inflammatory cells into the affected area.^13^ Desai et al.^16^ reported that patients treated with NPWT showed a greater reduction in wound size, earlier formation of granulation tissue, lower rates of infection, and higher satisfaction scores. Finally, NPWT reduces the need for frequent wound dressing changes.^2^ Extensive tissue loss may require skin grafting or flaps to achieve wound cover. Therefore, the plastic surgeon plays a vital role in the management of Meleney’s gangrene and should be involved early. Prompt and aggressive treatment is key to a successful outcome in patients with Meleney’s gangrene.

## 4. CONCLUSION

In patients with a suprapubic cystostomy, a history of a dislodged tube followed by skin changes should immediately raise suspicion for a deeper soft-tissue infection, with evolving Meleney’s gangrene as an important differential. Proper suprapubic catheter placement to avoid dislodgement and urinary leak into subcutaneous tissue planes cannot be overemphasized. Management should commence promptly with broad-spectrum antibiotics, wound debridement, and judicious wound care, with skin graft or flap as options for achieving wound cover. Our patient had a good recovery with wound healing by secondary intention following a failed skin graft.

## ACKNOWLEDGEMENT

We acknowledge all the doctors and nurses who participated in the patient’s management, particularly Dr Rabiu J Adamu.

## AUTHORS’ CONTRIBUTIONS

AMU: Study conceptualisation and writing the original draft. MMK: Supervision and substantially revised the manuscript. DJM: Formatted the figures and edited the manuscript. AK: Revised the manuscript. AOA: Data acquisition and writing the original draft.

## CONFLICT OF INTEREST

The author(s) declare that there is no conflict of interest.

## DISCLOSURE OF AI USE

The authors used DeepSeek and Grammarly solely to improve grammar and readability. No AI was involved in data analysis, clinical interpretation, or content generation. All authors take full responsibility for the final manuscript.

## DATA AVAILABILITY

The data cannot be publicly shared to protect the patient’s privacy but are available at the health records department of the institution.

## FUNDING

The author(s) received no financial support for the research, authorship, and/or publication of this article.

## ETHICAL APPROVAL AND CONSENT TO PARTICIPATE

Approval was waived as ethical approval was not required for publishing case reports at our institution.

## Figures and Tables

**Figure 1.**
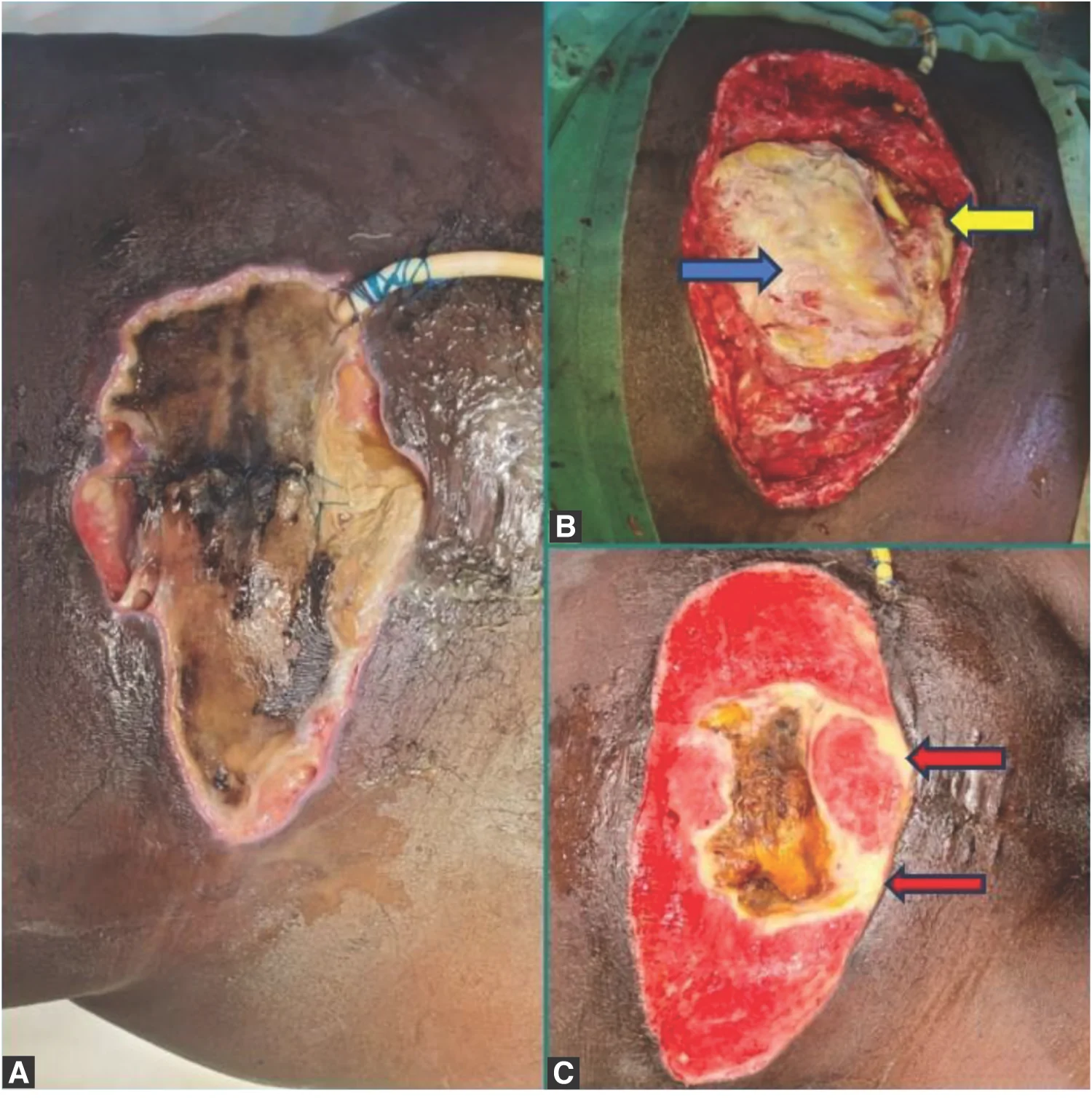
(A) Clinical photograph taken pre-debridement showing extensive gangrene of the lower abdominal wall with peri-vesical drain in situ. (B) Clinical photograph taken immediately post-surgical debridement showing extensive necrosis involving the skin, subcutaneous tissue, and rectus muscle. The exposed peritoneum (blue arrow) and urinary bladder (yellow arrow) with suprapubic tube in situ are covered by necrotic sloughs. (C) Clinical photograph taken 14 days post debridement showing the wound gradually granulating with some necrotic sloughs covering the peritoneum and pus (red arrow) emerging from the retropubic space.

**Figure 2.**
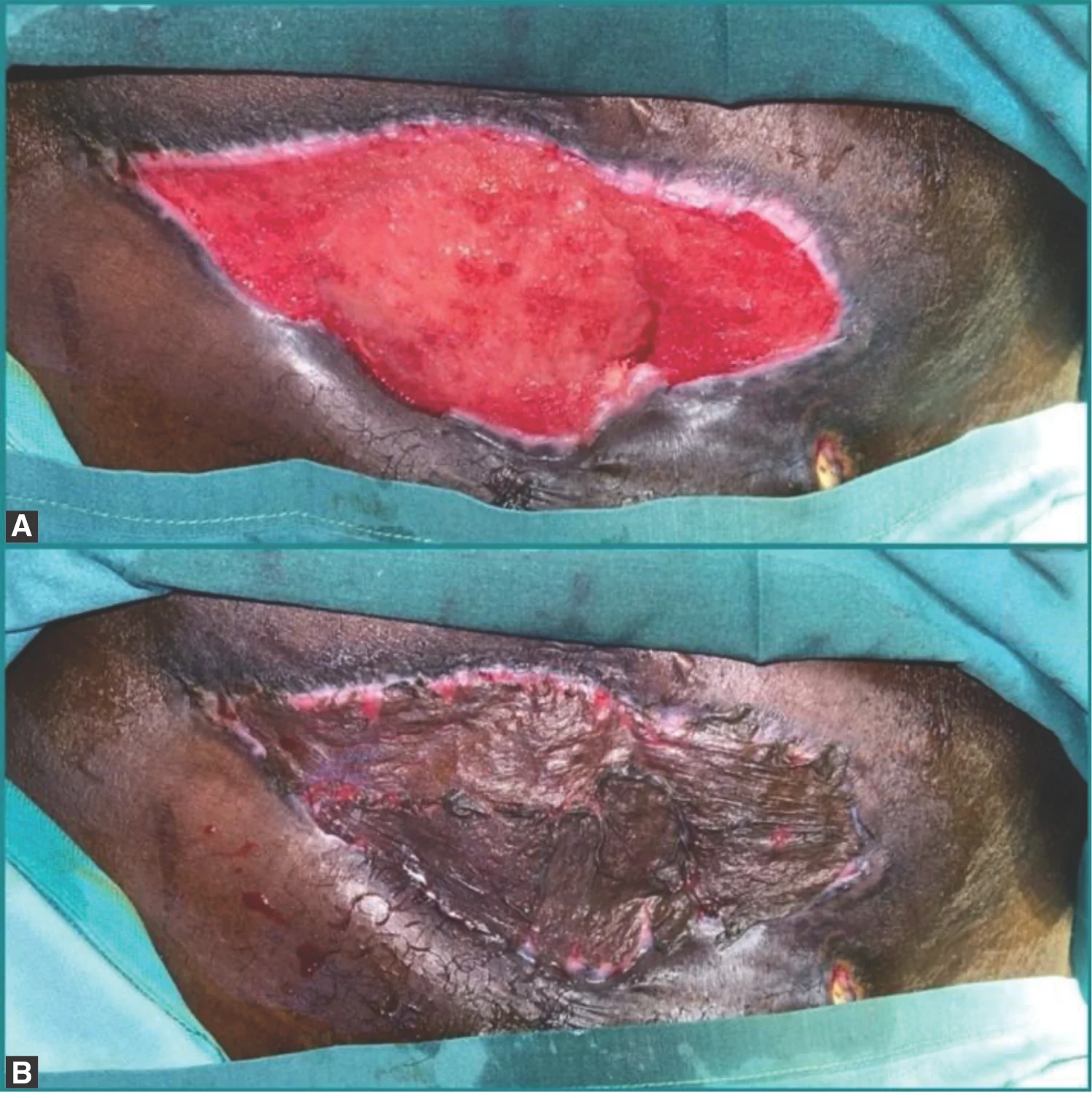
(A) Clinical photograph taken immediately before skin grafting showing wound covered by healthy granulation tissue. (B) Clinical photograph taken immediately following skin grafting.
